# Multidimensional Poverty as a Determinant of Techno-Distress in Online Education: Evidence from the Post-Pandemic Era

**DOI:** 10.3390/ijerph22070986

**Published:** 2025-06-23

**Authors:** Alejandro Cataldo, Natalia Bravo-Adasme, Juan Riquelme, Ariela Vásquez, Sebastián Rojas, Mario Arias-Oliva

**Affiliations:** 1 Escuela de Ingeniería Informática Empresarial, Facultad de Economía y Negocios, Universidad de Talca, Talca 3460000, Chile; acataldo@utalca.cl (A.C.); arvasquez16@alumnos.utalca.cl (A.V.); serojas16@alumnos.utalca.cl (S.R.); 2 Escuela de Ingeniería Comercial, Facultad de Economía y Negocios, Universidad de Talca, Talca 3460000, Chile; juriquelme@utalca.cl; 3 Departamento de Marketing, Facultad de Ciencias Económicas y Empresariales, Universidad Complutense de Madrid, 28040 Madrid, Spain; mario.arias@ucm.es; 4 Social and Business Research Lab, Departament Gestió d’Empreses, Universitat Rovira i Virgili, 43002 Tarragona, Spain

**Keywords:** multidimensional poverty, techno-distress, education, remote learning

## Abstract

The rapid shift to online education during the COVID-19 pandemic exacerbated mental health risks for students, particularly those experiencing multidimensional poverty—a potential contributor to psychological distress in digital learning environments. This study examines how poverty-driven techno-distress (technology-related stress) impacts university students’ mental health, focusing on 202 Chilean learners engaged in remote classes. Using partial least squares structural equation modeling (PLS-SEM), we analyzed multidimensional poverty and its association with techno-distress, measured through validated scales. The results suggest that poverty conditions are associated with 32.5% of technostress variance (R^2^ = 0.325), while techno-distress may indirectly relate to 18.7% of students’ dissatisfaction with academic life—a proxy for emerging mental health risks. Importance–performance map analysis (IPMA) identified housing habitability (e.g., overcrowding, inadequate study spaces) and healthcare access as priority intervention targets, surpassing purely digital factors. These findings indicate that techno-distress in online education may function as a systemic stressor, potentially amplifying pre-existing inequities linked to poverty. For educators and policymakers, this highlights the urgency of early interventions addressing students’ physical environments alongside pedagogical strategies. By framing techno-distress as a public health challenge rooted in socioeconomic disparities, this work advances preventive approaches to safeguard student well-being in increasingly hybrid educational landscapes.

## 1. Introduction

The 2030 Agenda identifies poverty eradication as the highest priority and a prerequisite for sustainable development [1]. While information technologies (ICTs) are widely recognized as tools to mitigate inequalities and poverty through improved access [2,3], the COVID-19 pandemic created an unprecedented natural experiment to test this paradigm. During global lockdowns, reliance on digital infrastructure—internet connectivity, devices, and remote applications—prevented economic collapse and enabled continuity in education and work. However, this crisis also exposed systemic vulnerabilities in equitable access to quality education, particularly in resource-constrained environments [4].

Disparities in technological access emerged as critical determinants of educational outcomes, exacerbating existing inequalities. Ref. [5] found that adolescents from socially disadvantaged backgrounds exhibited poorer academic performance, lower mental well-being, and a heightened risk of depression during the COVID-19 pandemic. Households lacking adequate software (e.g., productivity suites, PDF readers), peripheral devices (e.g., printers), or digital literacy skills faced compounded disadvantages during remote learning [6]. These inequities underscored how the digital divide transcends mere connectivity, amplifying pre-existing social inequalities [7] and reinforcing multidimensional poverty [8]. Notably, the pandemic highlighted techno-distress—stress induced by IT use [9]—as an understudied dimension of this divide, particularly among socioeconomically disadvantaged populations.

The existing literature establishes bidirectional relationships between poverty and technology access [8,10], socioeconomic status and stress [11,12], and family sociodemographic factors and educational outcomes [13]. However, the intersection of poverty, education, and techno-distress remains poorly characterized. While preliminary evidence suggests that income modulates techno-distress levels across welfare contexts [14], the mechanisms linking economic hardship to techno-distress in educational settings lack empirical validation. This gap is critical, as prolonged exposure to techno-distress may exacerbate educational attrition and perpetuate cycles of poverty.

This study addresses the question—to what extent does poverty affect techno-distress in students engaged in online learning? We analyze data from 202 Chilean university students using partial least squares structural equation modeling (PLS-SEM), a robust method for small-sample hypothesis testing [15]. By quantifying interactions between socioeconomic constraints and techno-distress, this work contributes to policy discussions on equitable digital education and sustainable development.

The remainder of this paper is organized into five sections. The next section summarizes the theoretical framework and the hypotheses of this study. Section 3 describes the materials and methods used. Section 4 presents the results obtained using PLS-SEM. In Section 5, our findings are discussed. Section 6 presents the main conclusions. The last section synthesizes the limitations and future directions of our work.

## 2. Theoretical Framework and Hypothesis Development

### 2.1. Definition and Measurement of Poverty

Poverty is defined as a deprivation of well-being, i.e., not being able to satisfy the basic needs of an individual or family [16]. Traditionally, poverty was measured through the use of the poverty line or income method, which involves determining a minimum income that is sufficient to meet basic needs [17]. From this perspective, the way to measure poverty involves determining a minimum income that is sufficient to meet basic needs [17]. However, this is a unidimensional measure of well-being and does not allow the researcher to capture different experiences of deprivation and poverty.

Over the last three decades, the concept of poverty has evolved into a more elaborate concept. Ref. [18] considers that poverty involves not only a lack of monetary resources to satisfy basic needs, but also the deprivation of basic human capabilities. People with low incomes tend to experience anxiety and somatization, mainly due to financial worries, job insecurity, or insecurity about the future [19]. Hence, poverty may have different meanings, such as poor health, low income, insufficient education, and food insecurity, among others [20]. Ref. [21] argues that, although income is one way of improving living conditions, it is not necessarily the best indicator of living conditions in itself.

Development researchers have proposed multidimensional poverty indices (MPIs) to capture the greater complexity of the concept of poverty [10]. The MPI analyzes two factors: the incidence, defined as the percentage of people that are poor, and the intensity, which corresponds to the average number of poverty dimensions in which poor people are deprived.

The literature contains a variety of opinions on the measurement of poverty. On the one hand, there are those who support measurement based on income, and who classify as poor a person whose income is below the poverty line [22]. On the other hand, there are those who use multidimensional poverty indices, and these are in turn divided into two groups. The first group considers that income poverty is complemented by multidimensional poverty, with a focus on non-monetary aspects [23], while the second considers that income is a dimension of multidimensional poverty, and that this is shaped in conjunction with education, health, and living standards [24,25].

Related to our research, we found several studies in favor of the use of MPI. One of these was put forward by [26], who uses the MPI proposed by [27] as a reference. In his paper, the author proposes an extension of a unidimensional index in the form of a two-dimensional approach, which was illustrated using data from Peru between 1998 and 2002 [26,28]. A second approach was proposed by [29], who presented a diffuse measure of vulnerability and applied the MPI proposed by [30]. A third approach is that introduced by [31], who presented a measure of vulnerability to multidimensional poverty, using the index developed by [20] as an indicator of poverty. A further approach was based on the MPI used by the Oxford Poverty and Human Development Initiative [32] at the University of Oxford, which was also developed using the counting methodology of [20].

### 2.2. Work–Home Conflict

The work–home conflict arises when the specific needs in one environmental dimension are in opposition with others [33,34]. This is a two-way interaction: the work demands produce conflicts in the household environment, and the household demands affect the work performance [35,36,37].

The work–home conflict is not dependent on a specific household or work role. Rather, it depends on a specific family and job demands [36,38]. For instance, a job emergency will negatively affect the fulfillment of household commitments, independent of the job type. The literature establishes that household and job environments are constantly competing for individual resources [39], inducing a trade-off between activities [40]. Notably, the COVID-19 pandemic shows that the work–home conflict, or its equivalent “study–home conflict”, also affects students attending remote classes. For instance, students reported losing a sense of control over multiple aspects of their lives, especially regarding where they lived and whether they or their family members would get sick [41].

As a concept, the work–home and home–work conflicts have evolved as the house and job environment do so [38]. In the traditional conception, the household was defined as a group comprising a working father, a housewife, children, and, in some cases, the elderly [37]. Today, the household concept has been adapted into a more flexible definition, including friends, housemates, grandchildren, and single parents [39].

Several authors agree that the work–home and home–work conflicts are the most important stressors of an individual’s performance, productivity, health, and satisfaction [9,42,43]. Because of this, it is necessary to deal with them appropriately to prevent stress or behavioral issues [36]. The home–work conflict (or home–study conflict) is also an important stressor for students in remote classes. During the pandemic, students reported feeling burnt out, as they suddenly felt that a much greater responsibility for learning had been put on their shoulders [41].

Considering that the work–life conflicts can be applied to any aspect that can cause similar results [37], in this paper, the work–home and home–work conflicts will be modified to the home–study conflict, because our unit of analysis are students.

In the present study, we adapt the term home–study conflict from the classic concept of work–home conflict, rather than using broader terms such as work–life interaction or work–life balance, due to its theoretical definition and applicability to the context of this research. This is primarily because work–home conflict focuses on the negative and bidirectional interference between the two domains (work and home) [1]. In contrast, work–life interaction refers to a continuous dynamic between the work sphere and other aspects of life, which may include both positive and negative effects [2,3]. Likewise, although the term work–life balance examines a desired state of harmony between both domains (work and life) [4,5], it does not accurately capture the conflict or stress that this study aims to analyze.

### 2.3. Definition and Dimensions of Techno-Distress

Technostress stems from constant interaction with ICTs in a specific environment. Technostress has been studied since 1984, and since then, its understanding has evolved, recognizing that the impact of technology is not exclusively negative but can also generate positive responses [7]. The recent literature has differentiated between two dimensions of technostress: techno-eustress and techno-distress. Techno-eustress refers to positive experiences derived from technology use, such as increased motivation, autonomy, well-being, efficiency, or job satisfaction [8,9]. On the other hand, techno-distress represents the negative side of technostress, characterized by anxiety, fatigue, exhaustion, frustration, or decreased performance caused by excessive or inappropriate technology use [7,10].

Techno-distress is derived from constant interaction with ICTs in a specific environment. The use of ICTs and the technological progress that drives them create a series of factors that are favorable to the development of techno-distress. These factors may arise when an individual’s employment requires the use of ICTs and involves a permanent connection to work, preventing the employee from disconnecting due to workload or working conditions [9,42]. Any mismatch in the prospects of ICT use in the work environment will trigger uncertainty in the individual, threatening his or her well-being and causing a physical and mental response. In other words, techno-distress is caused by demands related to ICT use that the individual is unable to meet, and with the presence of technological environmental conditions that the individual perceives as a negative effect [44].

The literature on techno-distress has identified five dimensions [43,45,46,47], which are as follows: techno-invasion, defined as the adverse effects of ICT in terms of invading the life of the user, creating situations that can affect the user at any time—this can generate performance problems both at work and in personal life due to a feeling of needing to remain constantly connected to ICT, which causes ambiguity and frustration [46]; techno-uncertainty, which refers to the uncertainty generated in an individual by constant changes and updates to the technology with which they interact, forcing them to be constantly studying [43]; techno-insecurity, which arises when an individual feels threatened by the ICT knowledge and skills of their peers, causing a feeling that they could lose their job [48]; techno-overload, the generation of information from ICT, whereby an individual is exposed to greater volumes of work and higher workloads due to the increasing use of technologies to improve communication, productivity, and employee performance [43]; techno-complexity, which occurs when, due to the needs and demands of the organization, an individual needs to constantly learn how to use new forms of ICT, or when an employee finds it difficult to learn how to use a new technology [48].

### 2.4. Satisfaction

Job satisfaction is the relationship between what an individual perceives of their work and what they really get [49]. Several studies have analyzed the relationship between techno-distress and satisfaction, mostly in workplace contexts. These studies have determined that techno-distress negatively affects job satisfaction [43,45,50]. The different causes of techno-distress result in lower labor commitment, high rotation levels, and lower satisfaction [42].

In the case of students, satisfaction with university life can be defined as an attitude resulting from their evaluation of the educational experience, services, or even physical facilities [11]. For students, the relationship between techno-distress and satisfaction has not been widely explored. Initial studies have identified that techno-invasion and techno-overload in online classes generate dissatisfaction with university life among students, which also reduces academic performance [12].

### 2.5. Theoretical Gap and Hypothesis Proposal

To date, no empirical study has investigated the relationship between multidimensional poverty—as conceptualized by the multidimensional poverty index (MPI)—and technostress among university students. Although some research has examined the association between socioeconomic disadvantages and mental health [13], these studies typically rely on unidimensional measures of poverty (usually income) and do not address the complex interplay of overlapping deprivations in education, health, housing, and access to services, as captured by the MPI [14].

Furthermore, prior literature on technostress in educational settings has largely focused on teachers [15] or generic student samples [16] without explicitly examining how structural vulnerabilities, such as multidimensional poverty, may shape the experience of technostress in post-pandemic hybrid learning environments.

To the best of our knowledge, no previous study has explored the combined impact of multidimensional poverty and role conflict arising from the merging of home and study domains on students’ technostress and subsequent well-being.

**Figure 1:**
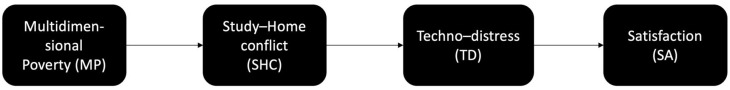
General model of this research.

Our study introduces a novel conceptual model integrating these elements and proposes three key postulates (Figure 1). First, poverty, measured multidimensionally, influences techno-distress. Second, the effect of poverty on techno-distress is mediated by the conflicting crossover between home and study tasks and responsibilities (study–home conflict). Finally, techno-distress reduces students’ satisfaction with their student life experience. Figure 1 shows a general diagram of these three postulates.

#### 2.5.1. Multidimensional Poverty and Study–Home Conflict

The causes of the work–home and home–work conflict have been widely analyzed [36,37,39]. Although the main causes of the home–work and work–home conflict have been identified, most of them have been analyzed under a workplace context, with no focus on students. Furthermore, multidimensional poverty has not been considered as a determinant of the work–home and home–work conflict. Therefore, our first hypothesis is as follows:

**H1.**  *Higher multidimensional poverty positively influences study–home conflict.*

Considering that the causes of the work–home conflict may be different that those that cause home–work conflict, we propose the following sub-hypotheses:

**H1a.**  *Higher multidimensional poverty positively influences study–home conflict.*

**H1b.**  *Higher multidimensional poverty positively influences home–study conflict.*

#### 2.5.2. Study–Home Conflict and Techno-Distress

The work/family border theory suggests that work (and in our case, study) and home are different domains, separated by a border. These domains are commanded by a more powerful actor and do not intermix or interrupt among themselves [33,34,42]. A child is also a student and does not possess much power in any of the domains. For this reason, they usually take up the demands of each domain. The student’s effort to balance and fulfill the demands of their parents and teachers generates the study–home and home–study conflict.

Students have been compelled to use information technologies to fulfill the demands of distance education and sometimes their household demands. This conflict can cause techno-distress. Thus, our second hypothesis is as follows:

**H2.**  *Higher study–home conflict positively influences techno-distress.*

Considering that the literature establishes that the effects of the work–home conflict may be different that the home–work conflict, we propose the following sub-hypotheses:

**H2a.**  *Higher study–home conflict positively influences techno-distress.*

**H2b.**  *Higher home–study conflict positively influences techno-distress.*

#### 2.5.3. Techno-Distress and Satisfaction with University Life

The literature suggests that techno-distress has several effects in workplace contexts, including a negative impact on end-user satisfaction and job satisfaction [51,52]. Some studies also indicate that techno-distress may impair students’ academic performance, adversely affecting the learning process. For instance, Ref. [53] examined how learners’ characteristics—such as digital literacy, self-directed learning, motivation, and perceived stress—influenced academic engagement in remote classes. Their results confirmed the moderating role of perceived stress. Additionally, Ref. [54] found significant positive associations between academic stress and problematic TikTok use, which may contribute to university dropout [55]. Based on these findings, we propose the following hypotheses:

**H3.**  *Higher techno-distress negatively influences satisfaction with university life.*

**Figure 2:**
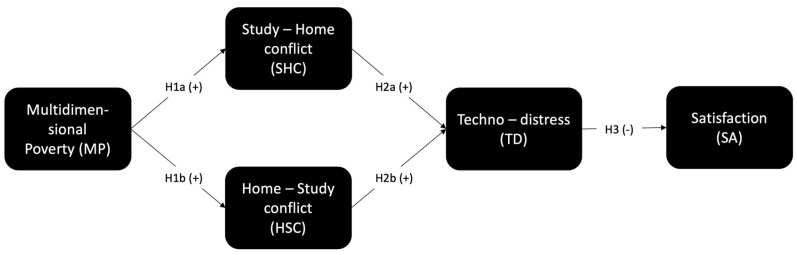
Model of the proposed hypotheses.

Figure 2 shows a schematic diagram of the theoretical model studied.

## 3. Materials and Methods

Data collection was carried out using a self-administered online questionnaire. The design of the questionnaire incorporated questions validated in previous research, and these are summarized in Table 1.

The questions were translated into Spanish and adapted for our purposes, taking care not to change the original meaning. The techno-distress questionnaire originally contained 23 questions, 5 of which were related to the dimension of techno-insecurity in the context of employment. Since the students were not in an employment situation, we decided not to use these questions. This decision is consistent with previous adaptations of the scale in non-working populations. For example, ref. [17] adapted the scale for educational samples without including the techno-insecurity dimension, given its limited applicability to non-work-related educational settings. By omitting these items, we maintain consistency with the existing research on student-centered technostress measurement.

It is worth noting that MPI is a composite construct for which the indicators are defined differently by each government. There is therefore no standardized scale for multidimensional poverty; instead, measurements depend on what each country has established as the relevant indicators and the dimensions necessary to measure it. This methodological flexibility is consistent with the approach proposed by [18], whose multidimensional poverty measurement framework explicitly allows countries to define their own context-sensitive dimensions and indicators. We decided to use questions elaborated by the government of Chile for three reasons. Firstly, the sample of this study consists of Chilean students. Secondly, the Chilean MPI, developed by the Ministry of Social Development and Family [19], includes dimensions such as education, health, employment, and housing—dimensions that are broadly aligned with those used in other international applications [20]. This conceptual overlap provides some support for potential comparability, despite contextual specificity. Secondly, the MPI items included by the Chilean government in its measurement are widely used and accepted in the related literature. Therefore, it seemed to us that the Chilean multidimensional poverty questionnaire could also be applicable to other countries. Thirdly, the Chilean scale corresponds to an instrument that has been used for several years to measure poverty in that country. Thirdly, the Chilean MPI has been used systematically in national surveys, such as the CASEN survey, since 2013, ensuring psychometric consistency and institutional legitimacy over time [19].

According to the government of Chile, the MPI is divided into five dimensions, related to education, health, labor and social security, social networks and cohesion, and housing and the environment [56]. Each of these dimensions had three indicators, which were originally measured with one question each; however, since the questions were very extensive in some cases, we separated them into two or three questions for ease of understanding by the respondent.

The survey consisted of 53 questions (see Table A1 in Appendix A), of which 21 referred to multidimensional poverty, 18 to techno-distress, 5 to home–study conflict and study–home conflict, and 4 to satisfaction with university life [57]. In addition, three security questions were incorporated to verify that the respondent had answered correctly and to prevent common method bias. The responses to the MPI questions were measured with dummy variables based on whether deprivation was present (1) or not (0) for each indicator. The responses to the home–study conflict, study–home conflict, and techno-distress questions were measured on a five-point Likert scale (from “Strongly disagree” to “Strongly agree”). The responses to the satisfaction with university life questions were measured on a seven-point Likert scale (from “Extremely disagree” to “Extremely agree”).

**Table 1:** Number of questions by construct and reference source.

Construct	Dimension (Second-Order Construct)	Number of Questions	Code	Reference Source
**Satisfaction with university life**	(First-order construct)	04	SA	[21]
**Home–study conflict**	(First-order construct)	05	HSC	[22]
**Study–home conflict**	(First-order construct)	05	SHC	[22]
**Multidimensional poverty**	Education	03	EDb	[19]
	Health	04	HEb	
	Work and social security	03	WOb	
	Networks and social cohesion	05	NTb	
	Housing and environment	06	HAb	
**Techno-distress**	Technological overload	05	OL	[23,24,58]
	Technological invasion	04	IN	
	Technological complexity	05	CO	
	Technological uncertainty	04	UN	

Twelve pre-tests were administered to the students, who were contacted via videocall. Based on the corrections and concerns expressed by the participants, several modifications were made to the questionnaire. Most of these adjustments were made to clarify the questions. The responses of the participants to the pre-test were excluded from the final sample.

Once the corrections derived from the pre-test were completed, the data collection process began on 19 July 2021, by sending an online survey to the students enrolled in diverse postsecondary institutions, including vocational schools (also termed trade schools), technical colleges, and universities. The survey started with the presentation of informed consent, which participants had to accept in order to proceed with the rest of the questionnaire. Data collection continued until 13 August 2021, resulting in a total of 202 cases that were retained for statistical analysis.

This study was reviewed and approved by the relevant ethics committee on 10 July 2021. The process complied with all institutional and international ethical standards. Prior to participating in the online survey, all participants received a digital informed consent form detailing the following: the study objectives, the voluntary nature of participation, guarantees of anonymity and data confidentiality, as well as options to accept or decline participation. This process strictly adhered to the principles established in the Helsinki Declaration.

For the analysis, we used a second-order structural equation model (PLS-SEM). According to [58,59], PLS-SEM should be used when a small population means that the sample size is restricted, and distribution issues are a concern, such as a lack of normality.

The techno-distress construct (TD) was modeled as a high-order reflexive construct [47], consisting of four first-order constructs, corresponding to the four dimensions of technostress: techno-overload (OL), techno-invasion (IN), techno-complexity (CO), and techno-uncertainty (UN). The construct of multidimensional poverty (MP) was also modeled as a high-order construct, and consisted of five dimensions: education (EDb), health (HEb), housing (HOb), social support networks and social cohesion (NTb), and work (WOb). Multidimensional poverty was modeled as formative, since this construct is a pragmatic elaboration or composite construct, i.e., the five dimensions mentioned are considered causes of multidimensional poverty [60].

According to the two-stage approach [59], the analysis of the model was performed in two phases: repeated indicators and the evaluation of the second-order model. In the first stage, the two second-order constructs (techno-distress and multidimensional poverty) are created based on the manifest variables of the underlying dimensions, which are taken as first-order constructs. The items corresponding to MPI and techno-distress were used twice: firstly, each item was used in the low-order constructs, and secondly, each item was used to form the high-order constructs. In the second stage, we used the latent variable scores as manifest variables in the high-order measurement model.

## 4. Results

We analyzed the results using the PLS method with SmartPLS, specifically version 3 of the software.

### 4.1. Sample Characteristics and Common Method Bias

Table A2 in Appendix A presents the main descriptive results by gender. The poverty indicators have been averaged by dimension.

Two separate criteria suggest that the sample is large enough for evaluation. Firstly, the sample size exceeds the criterion established to achieve a statistical power of 80% for detecting R^2^ values of at least 0.10 with a 1% significance (169 cases) [61]. Secondly, Ref. [15], based on the inverse square root method, suggests a size sample of 155 cases when minimal path coefficients are expected to be between 0.110 and 0.200. The size of the sample exceeded these two thresholds.

To reduce the risk of common method bias, we took actions before and after data collection. Before applying the questionnaire, we took two preventive actions: we used different scale measures for the different constructs and incorporated three security questions into the questionnaire. After data collection, we checked for common method bias, running a Harman’s single-factor test. The results confirmed that there was no problem with common method bias in the data, since the total variance extracted by one factor was 21.5%, which was less than the recommended threshold of 50% [25].

### 4.2. General Model

Techno-distress and multidimensional poverty were modeled as second-order constructs. The study–home conflict, home–study conflict, and satisfaction with university life were modeled as first-order constructs.

We started by adjusting the techno-distress. We first examined all reflexive constructs based on the factor loadings (≥0.6 for exploratory studies), composite reliability (≥0.7), and average variance extracted, AVE (>0.5). See Table A3 in Appendix A [26].

In the cases of study–home conflict, home–study conflict, and satisfaction with university life, all indicators showed a good level of convergent validity and reliability (SHC_1 was the item having the lowest load with 0.796); see Table A3.

Next was the reliability and validity of multidimensional poverty (see Table A3). The multidimensional poverty assessment followed recommendations for formative-formative constructs [61]. No multidimensional poverty indicator exceeded the acceptable limit of a VIF (the VIF ranged between 1.009 and 4.173, <5.0). Four indicators had weights and loadings that were not significant, MP-EDb-3, MP-HEb-1, MP-HOb-2, and MP-NTb-1, and they were removed. The MP-WOb-3 indicator was removed due to variance problems.

As the reader can see in Table A4, techno-distress loadings are above 0.6 and multidimensional poverty’s weights are significant. AVE is 0.598, indicating convergent validity. Composite reliability is 0.855 and Cronbach’s alpha is 0.772, indicating internal consistency reliability.

The discriminant validity was assessed using the heterotrait–monotrait ratio criterion (HTMT) (<0.90). Table A5 shows estimates for all reflexive constructs, and it can be seen that all indicators are within the acceptable range, thus confirming the reliability and validity of the outer model.

Since the results provided support for the reliability and validity of the first-order measurement model [61], we advanced to the second stage of the analysis. At this stage, the first-order scores served as manifest variables in the two second-order constructs of the measurement model. Table A6 shows the validity and reliability estimates for the second-order model. As can be seen, all constructs are within the recommended thresholds, showing that the outer model meets the validity and reliability requirements.

After evaluating the outer model, we assessed the structural model by implementing a bootstrapping method (5000 sub-samples, Bca bootstrap, two-tailed, 5% level of significance, factor weighting scheme). Table 2 summarizes the main results for the structural model. The coefficients of determination (R^2^) and the significance levels of the path coefficients were used to validate the structural model [26].

**Figure 3:**
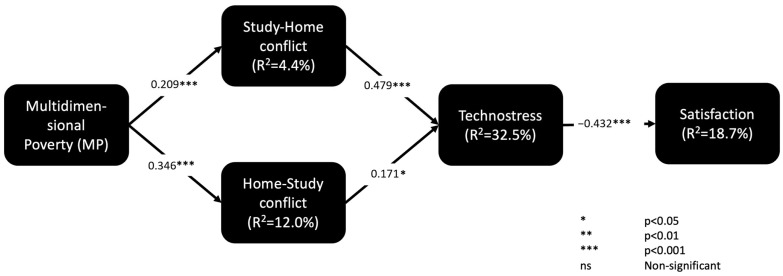
Final model with the results of the hypothesis analysis.

Figure 3 shows the final model with the hypothesis analysis.

At first glance, the effect size *f*^2^ indicates that home–study conflict has a relatively high influence on techno-distress (see Table 2). Similarly, techno-distress has a significant effect on student satisfaction in the context of online classes. The R^2^ analysis also demonstrates that the combined effect of the conflict between home and studies is substantial, suggesting that this conflict is a significant cause of techno-distress in students. Although the effect of techno-distress on satisfaction is moderate, it should not be underestimated. Student satisfaction is a mental state influenced by numerous factors. The negative contribution of techno-distress by 18.7% suggests its considerable influence.

The Q^2^ value (Stone–Geisser’s) indicates that the model’s out-of-sample predictive power has predictive relevance for the four endogenous constructs under consideration [61].

### 4.3. Post Hoc Analysis: Importance–Performance Map Analysis

We wanted to better understand the effect of each dimension of poverty on techno-distress and propose measures for action. Therefore, we performed an importance–performance map analysis (IPMA). IPMA aims to offer information about the role of the antecedent constructs of a target construct and their relevance for managerial actions [62]. In an IPMA, the total effects, which represent the importance of the predecessor constructs in terms of shaping a certain target construct, are contrasted with their average latent variable scores indicating their performance. This information could be used by a manager to make managerial decisions. For example, when a manager needs to improve a target construct, they should focus on predecessors with a relatively high importance and a relatively low performance [59,63]. In our case, we wanted to identify which specific multidimensional poverty indicators had high importance and high performance. We looked for the high-performing indicators because they are the ones that most increase techno-distress, i.e., they have an undesirable effect and should be reduced.

**Figure 4:**
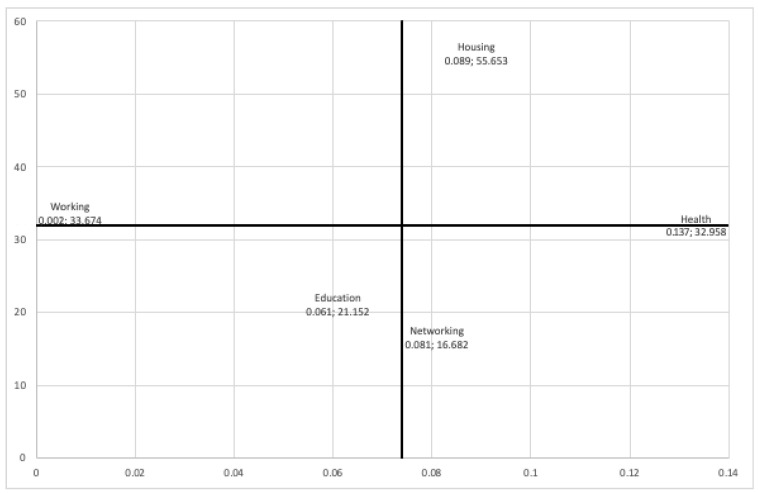
IPMA map showing indicators (i.e., dimensions of poverty) level for the multidimensional poverty construct.

Refs. [59,63] recommend running an IPMA at the indicator level for formative constructs, as this can help the researcher gain more specific information on how to increase performance for a desired target construct or decrease it for an undesired one. So, we turn our attention toward the indicators of multidimensional poverty. The IPMA map to the indicator level is shown in Figure 4. The vertical line cuts the horizontal axis at the average value of the importance of the indicators (0.074), while the horizontal line cuts the vertical axis at the average value of the performance of the indicators (32.02).

As can be seen in Figure 4, there were two dimensions with high importance and high performance, which were housing and health. This tells us that to decrease the aggregated effect of multidimensional poverty on techno-distress, it is necessary to improve housing and neighborhood conditions, on the one hand, and to improve the health coverage of the members of the student’s household, on the other.

## 5. Discussion

The main purpose of this research was to study how multidimensional poverty and study–home conflict affect techno-distress in students due to the need to take remote classes. To do this, we analyzed whether or not multidimensional poverty influenced conflict between study and home and techno-distress. Also, we analyzed whether or not techno-distress decreased satisfaction with university life experience in students. The results confirmed our hypotheses: study–home conflict had a significant effect on techno-distress. Multidimensional poverty was significantly associated with study–home conflict, suggesting a potential indirect pathway to techno-distress. In addition, techno-distress was found to influence students’ university life satisfaction due to the need to take remote classes.

### 5.1. Effects of Multidimensional Poverty on Study–Home Conflict

The results show that multidimensional poverty influences the study–home conflict and the home–study conflict. These results can be explained by the online class environment. In online classroom contexts, low-income students are the most harmed. They do not have the infrastructure or the technology needed to undertake the online teaching methods. In many cases, they shared limited household spaces with other family members, with an increase in their interactions that may lead to conflict. Furthermore, many household heads were not in a stable job position. The anxiety generated by such a situation passed through the household, generating conflict [64]. This coincides with the family stress model, in which a low-income, economic difficulties, and anxiety produce conflict and stress in the household heads, which is transmitted to their children [65,66].

### 5.2. Effects of Study–Home Conflict on Techno-Distress

Our results indicate that the conflict between study and home has a positive effect on techno-distress levels. These results make sense considering that online classes pass the study domain directly to the home domain, disrupting the daily interactions and generating new demands. This is consistent with the work/family border theory [34], which states that interaction between domains is the main source of conflict. The results are in line with the current literature in a workplace context, where there is a consensus that the work–home conflict is one of the main drivers affecting techno-distress [39,42,43,46]. Additionally, the results align with [41], which found that the self-regulation required by online classes is impacted by home demands. However, these findings contradict those of [67], who reported no significant levels of techno-distress among teachers in remote classes. Notably, few studies have explored the impact of study–home conflict on techno-distress in students, highlighting the need for further research.

While our model suggests possible indirect effects from multidimensional poverty to techno-distress via study–home and home–study conflict, it is important to note that no formal mediation analysis was conducted in this study. Future research should formally test these mediation pathways, ideally using longitudinal data to better assess the causal relationships between these variables. This would strengthen the understanding of how multidimensional poverty exacerbates techno-distress through domain conflicts.

The results show that home–study conflict had a relatively weak effect on technostress, whereas its counterpart, study–home conflict, exhibited one of the strongest effects in the model. This discrepancy may raise questions regarding the explanatory value of maintaining these two constructs as distinct dimensions, and whether they are empirically distinguishable.

Two main arguments support keeping WSC and SWC as separate constructs. First, from a theoretical perspective, Ref. [2] argues that the psychological and emotional dynamics associated with each type of conflict differ depending on whether the source originates in work or in family life, triggering distinct cognitive, affective, and behavioral responses. When work interferes with family life, individuals may experience accumulated stress due to long working hours, feelings of guilt for not being present with their families, mental and emotional exhaustion, and frustration when failing to meet important family responsibilities. Conversely, when family interferes with work (or study), individuals may feel anxiety over pending family matters, distraction during the workday, internal pressure to resolve family issues, and fear of judgment from colleagues or supervisors. Clark emphasizes that these two types of conflict are not simply inverses of each other; rather, they have different causes, consequences, and coping mechanisms. This distinction is essential for understanding how each direction of conflict uniquely impacts outcomes such as technostress. Second, from an empirical standpoint, evidence also supports the differentiation between the two constructs. The HTMT index, which assesses discriminant validity in structural equation modeling, yielded a value of 0.449 between SHC (study–home conflict) and HSC (home–study conflict), well below the critical threshold of 0.85/0.90 recommended by [27]. This indicates that, although the two constructs are related, they clearly differ in empirical terms. This aligns with previous studies, such as [22], which found moderate correlations between both dimensions without reaching levels of redundancy that would justify merging them into a single construct. Therefore, from both a theoretical and practical perspective, it is relevant to maintain them as separate constructs. Combining them could obscure meaningful effects and reduce the explanatory power of the model, especially considering that their impact on variables such as technostress may be differential.

### 5.3. Effects of Techno-Distress on Satisfaction with University Life

The present research confirms that techno-distress negatively affects university students’ satisfaction. Specifically, techno-distress caused by online classes affects students’ expectations about their university life. This result is in line with the literature on the effects of techno-distress in work contexts, in which researchers have also suggested that techno-distress can lead to lower productivity, performance, and satisfaction [43,50]. In student contexts, research on the effect of techno-distress is limited. However, authors have concluded that student engagement was more adversely affected among schools located in areas with lower coverage of high-speed broadband [68] and that techno-distress can negatively affect student satisfaction [41,44].

It is worth noting that the results show that techno-distress negatively affected student satisfaction by 18.7%. Although this effect is moderate, its relative importance is high. Student satisfaction is influenced by various factors, such as instructional support, technology acceptance, and perceived presence, among others [69]. Therefore, an effect of 18.7% on satisfaction is considerable relative to other factors and merits the attention of institutions and educators.

### 5.4. IPMA and Implications

Our results are consistent with a model where multidimensional poverty is associated with higher techno-distress, both directly and potentially indirectly through the study–home conflict.

The map in Figure 4 shows that two dimensions are of high importance and have the greatest impact on techno-distress: health and housing.

An ineffective healthcare system will likely cover the family of a poor student in a low-income country. Such a system is characterized by a failure to provide appropriate healthcare or long waiting times for medical care. This inequality exacerbates the sense of poverty in most of the population. This would also be in line with Sapolsky’s conclusion about stress not being determined by “being poor”, but by “feeling poor” [12].

On the other hand, a lack of habitability can be caused by two situations: living in an overcrowded home, or in a house with poor infrastructure. A student living in an overcrowded home will have evident problems connecting to classes: they will probably not have a dedicated study space suitable for connecting to online classes, or they will be distracted and unable to pay attention to the class by the noises of people talking, appliances running, or TVs on, among others. This in line with [68], who found a relationship between infrastructure at home and student engagement.

Hence, policies aimed at improving habitability conditions and investments in neighborhoods could help decrease students’ techno-distress in distance classes. Secondly, living in a household where some or all its members do not have healthcare system coverage or cannot access timely medical care increases techno-distress. Consequently, improving healthcare system coverage will decrease techno-distress in students due to the need to take remote classes.

Furthermore, IPMA maps confirm that external factors related to education should be considered when implementing distance education. This is consistent with [69], who concluded that ‘A focus on educational policy and schools alone will not in itself address the poverty-related attainment gap. What is required is a holistic focus on public policy, informed by interdisciplinary research, and a focus on building a strong infrastructure of support around schools, families and communities’.

## 6. Conclusions

This study suggests an association between multidimensional poverty—particularly inadequate housing and healthcare access— and higher levels of techno-distress in online learning (R^2^ = 32.5%). The analysis indicates that these factors may indirectly contribute to student dissatisfaction with academic life (explaining 18.7% of its variance), which could signal emerging mental health risks. These findings highlight potential pathways through which socioeconomic inequities might exacerbate technology-driven stressors, creating systemic barriers to psychological well-being in educational settings. While mediation was not formally tested, the results identify housing habitability (e.g., overcrowding, unsafe neighborhoods) and healthcare gaps as potential intervention targets. This perspective underscores that techno-distress is not merely a pedagogical challenge but may also reflect a public health issue rooted in structural disparities.

For educators and mental health practitioners, these results highlight the urgency of early, preventive interventions tailored to students’ lived realities. Schools and universities must adopt trauma-informed approaches that (1) integrate mental health screenings to identify at-risk students in online/hybrid programs; (2) collaborate with local governments to improve housing conditions and expand healthcare access for low-income families; and (3) train educators to recognize techno-distress symptoms linked to socioeconomic deprivation.

Policymakers should prioritize school–community partnerships that address environmental determinants of mental health, aligning with the WHO’s call for intersectoral action in education. Examples include subsidized study spaces in marginalized neighborhoods and campus-based health clinics.

As blended learning becomes permanent post-pandemic, mitigating techno-distress demands systemic reforms that bridge education, urban development, and healthcare—key to safeguarding students’ mental well-being in an era of digitized inequities.

### Limitations and Future Directions

This study’s generalizability is limited by several factors. First, it focused exclusively on Chilean university students and employed a multidimensional poverty index based on national indicators, which may constrain the applicability of the findings to other countries or cultural contexts. Second, the cross-sectional design restricts the ability to draw robust causal inferences between poverty, techno-distress, and academic outcomes. Third, all data were collected via self-reported questionnaires, which are susceptible to social desirability and response biases. Fourth, one dimension of the technostress construct—techno-insecurity—was excluded from the analysis, potentially compromising the comprehensiveness of the measurement. Fifth, the model did not account for other relevant factors such as digital skills, personality traits, or social support, which may also significantly influence techno-distress and academic satisfaction.

Future research should aim to validate these findings in more diverse cultural and educational contexts using standardized, internationally comparable measures of multidimensional poverty. Longitudinal designs are particularly encouraged to clarify causal pathways. Moreover, incorporating a fuller range of technostress dimensions and additional psychosocial variables would enhance the explanatory power and external validity of future models.

Despite these limitations, our work provides empirical evidence that techno-distress in digital education is inextricable from broader social determinants. Addressing this challenge demands cross-sectoral collaboration, ensuring that technological advancements in education do not inadvertently exacerbate inequalities but instead contribute to sustainable, equitable development.

## Figures and Tables

**Table 2:** Main results for the structural model: hypothesis assessment, coefficient of determination, size effect, and predictive validity.

Relationship	Hypothesis Assessment		Construct Effect Size	
	Path	Supported?	f^2^	Effect
**MP → SHC (H1a)**	0.209 ***	Yes	0.046	Small
**MP → HSC (H1b)**	0.346 ***	Yes	0.136	Small
**SHC → TS (H2a)**	0.479 ***	Yes	0.285	Medium
**HSC → TS (H2b)**	0.171 *	Yes	0.036	Small
**TD → SA (H3)**	−0.432 ***	Yes	0.230	Medium
**Endogenous variable**	**Coefficient of determination (R^2^)**	**Effect**	**Predictive relevance (Q^2^)**	**Predictive relevance established (Q^2^ > 0)?**
**Study–home conflict (SHC)**	0.044	Weak	0.029	Yes
**Home–study conflict (HSC)**	0.120	Weak	0.080	Yes
**Techno-distress (TD)**	0.325	Substantial	0.167	Yes
**Satisfaction with university life (SA)**	0.187	Moderate	0.143	Yes

*: *p* < 0.05; **: *p* < 0.01; ***: *p* < 0.001; ns: non-significant

## Data Availability

The data that support the findings of this study are available on request from the corresponding author.

## Appendix A

**Table A1:** Spanish questionnaire and translation.

Construct	Spanish	Translation
Income level	¿Cuánto es el ingreso total promedio mensual de tu hogar?	What is the average total monthly income of your household?
Multidimensional Poverty	¿Cuántos de los integrantes de su hogar de entre 4 a 18 años de edad no está asistiendo a clases presenciales u online ni ha egresado de cuarto medio?	How many of the members of your household between 4 and 18 years of age are not attending classes in person or online and have not graduated from high school?
	¿Cuántos de los integrantes de su hogar menores a 22 años, actualmente estudiando, está atrasado dos años o más con respecto al curso que le corresponde de acuerdo con su edad?	How many of the members of your household under 22 years of age, currently studying, are two years or more behind the course that corresponds to their age?
	¿Cuántos de los integrantes de tu hogar mayores a 18 años no terminó el cuarto medio ni está actualmente estudiando?	How many of the members of your household older than 18 years old did not finish high school and are not currently studying?
	¿Cuántos de los integrantes de tu hogar de entre 0 y 6 años con sobrepeso u obesidad, o está en desnutrición o riesgo de desnutrición?	How many of the members of your household between 0 and 6 years old are overweight or obese, or malnourished or at risk of malnutrition?
	¿Cuántos de los integrantes de tu hogar no están afiliados a un sistema previsional de salud (por ejemplo, Fonasa o Isapre) y no tiene otro seguro de salud?	How many of the members of your household are not affiliated to a health insurance system and do not have other health insurance?
	En los últimos doce meses ¿Cuántos de los integrantes de tu hogar pensaron en pedir atención de salud, pero no la consiguieron porque no tuvieron dinero, como poder llegar al lugar de atención, o no les dieron hora?	In the last twelve months, how many of the members of your household thought about seeking health care, but did not get it because they did not have money, how to get to the place of care, or they were not given an appointment?
	En los últimos doce meses ¿Cuántos de los integrantes de tu hogar necesitaron tratamiento médico por enfermedad cubierta por el seguro AUGE o GES pero no la recibieron?	In the last twelve months, how many of the members of your household needed medical treatment for an illness but did not receive it?
	En los últimos doce meses ¿Cuántos de los integrantes de tu hogar mayores de 18 no tiene trabajo y ha buscado uno?	In the last twelve months, ¿how many of the members of your household over 18 do not have a job and have looked for one?
	¿Cuántos de los integrantes de tu hogar mayores de 15 años que se encuentra trabajando no cotizan en alguna AFP ni es trabajador independiente?	How many of the members of your household over 15 years of age who are working do not contribute to a pension fund and are not self-employed?
	¿Cuántos, de los integrantes de tu hogar mayor a 60 años, si es mujer, o 65, si es hombre, no recibe pensión, jubilación u otro ingreso por arriendos, retiro de utilidades, dividendos e intereses?	How many of the members of your household over 60 years of age, if female, or 65, if male, do not receive a pension, retirement or other income from rents, withdrawal of profits, dividends and interest?
	Fuera de los miembros del hogar ¿Cuantos de sus integrantes no cuentan con ninguna persona que pueda ayudar en situaciones relevantes de apoyo o cuidado?	Out of the members of the household, how many of its members do not have any person who can help in relevant situations of support or care?
	En los últimos 12 meses ¿Cuántos de los integrantes de tu hogar mayores de 13 han participado en alguna organización o grupo social?	In the last 12 months, how many of your household members over the age of 13 have participated in any organization or social group?
	¿Cuántos de los integrantes de tu hogar mayores de 18 años ocupados pertenecen a alguna organización relacionada con su trabajo?	How many of your employed household members over the age of 18 belong to an organization related to their work?
	¿Cuántos de los integrantes de tu hogar ha sido discriminados o tratados injustamente por ser extranjeros, su identidad de género, sus creencias religiosas, ideología política u otros?	How many of the members of your household have been discriminated against or treated unfairly because of their foreign nationality, gender identity, religious beliefs, political ideology or other?
	Durante el último mes ¿Cuántos de los integrantes de tu hogar han siempre vivido o presenciado tráfico de drogas o balaceras?	During the last month, how many of the members of your household have ever experienced or witnessed drug trafficking or shootings?
	¿Cuántos dormitorios tiene tu casa?	How many bedrooms does your household have?
	Incluyéndote ¿Cuántas personas en total viven en tu casa?	Including yourself, how many people in total live in your home?
	¿Tiene tu casa alguna de las siguientes condiciones estructurales? Si no cumple ninguna de ellas, por favor marca la última opción. - Muros en mal estado - No tiene piso o está en mal estado - Hay goteras o el techo está en mal estado - La vivienda en general podría considerarse en mal estado - Ninguna de las anteriores	Does your home have any of the following structural conditions? If it does not meet any of them, please check the last option. - Walls in poor condition - No floor or in poor condition - Leaks or roof in poor condition - The house in general could be considered in poor condition - None of the above
	¿Tiene tu casa alguna de las siguientes condiciones sanitarias? Si no cumple ninguna de ellas, por favor marca la última opción. - Baño WC con alcantarillado o fosa séptica - Llave de agua dentro de la vivienda - Agua potable - No tiene ninguna de las anteriores	Does your home have any of the following sanitary conditions? If none of the above, please check the last option. - Bathroom WC with sewer or septic tank - Water faucet inside the house - Drinking water - None of the above
	¿En tu el área de residencia ocurre alguna(s) de las siguientes condiciones? Si no cumple ninguna de ellas, por favor marca la última opción - Hay frecuentemente dos o más problemas de contaminación medioambiental (ruidos fuertes, basura, smog, contaminación lumínica, otros) - Carece de servicios de salud, educación o transporte - Ninguna de las anteriores	Do any of the following conditions occur in your area of residence? If none of the above, please check the last option. - There are frequently two or more environmental pollution problems (loud noise, garbage, smog, light pollution, other). - Lacking health, education or transportation services - None of the above
	¿Cuántas personas de tu hogar usan transporte público o no motorizado y en promedio demoran una hora o más en llegar desde su vivienda al lugar de su trabajo principal?	How many people in your household use public or non-motorized transportation and on average it takes them an hour or more to get from their home to their primary work location?
**Economic hardship**	Durante el último año: ¿Qué tan a menudo, tu o en tu casa, han tenido problemas para pagar las cuentas de servicios básicos?	During the last year, how often did you…: …have trouble paying the bills,
	¿Qué tan a menudo, tu o en tu casa, no han tenido dinero suficiente para comprar productos básicos como comida, ropa u otros?	…not have enough money to buy food, clothes, or other household goods,”
	¿Qué tan a menudo, tu o en tu casa, no han tenido medios económicos para pagar por atención médica o comprar medicamentos?	…not have enough money to pay for medical care.
**Technodistress**	Las plataformas de estudios online me obligan a estudiar más rápido.	Online study platforms force me to study faster.
	Las plataformas de estudios online me obligan a estudiar más de lo que puedo manejar	Online study platforms force me to study more than I can handle.
	Las plataformas de estudios online me obligan a estudiar en tiempos muy acotados	Online study platforms force me to study in very limited time frames.
	Las plataformas de estudios online me obligan a estudiar más rápido	Online study platforms force me to study faster.
	Me veo obligado a cambiar mis hábitos de estudios para adaptarme a las tecnologías	I am forced to change my study habits to adapt to technologies
	Tengo una mayor carga de estudio debido a que las tecnologías de estudios online son más complicadas	I have a heavier study load because the online study technologies are more complicated.
	Debido al estudio online, tengo que mantenerme en contacto con mis compañeros o profesores, incluso en periodos que no hay clases	Due to online study, I have to keep in touch with my classmates or professors, even in periods when there are no classes.
	Debido al estudio online, tengo que sacrificar tiempo de mis vacaciones y fines de semanas.	Due to online study, I have to sacrifice time from my vacations and weekends.
	Siento que mi vida personal está siendo invadida por los estudios online	I feel that my personal life is being invaded by online studies.
	No sé usar bien las tecnologías de estudio online para hacer bien mis tareas	I don’t know how to use online study technologies well to do my homework well.
	Necesito más tiempo para poder entender y usar las tecnologías de estudio online	I need more time to be able to understand and use online study technologies.
	Me cuesta tener tiempo suficiente para mejorar mis habilidades en el uso de las tecnologías de estudio online	I find it hard to have enough time to improve my skills in using online study technologies
	Encuentro que mis compañeros más jóvenes saben más sobre el uso de tecnologías de estudio online que yo	I find that my younger peers know more about using online study technologies than I do
	Frecuentemente, encuentro que para mí las tecnologías de estudio online son demasiado complejas de entender y usar	Frequently, I find that for me online study technologies are too complex to understand and use
	Siempre hay nuevas actualizaciones en las tecnologías de estudios online	There are always new updates in online study technologies
	Constantemente, hay cambios en los softwares que se usan en mi universidad o instituto para los estudios online	Constantly, there are changes in the software used at my university or institute for online studies
	Tengo que constantemente estar cambiando el hardware (computador, teléfono, tablet u otros) para los estudios online	I have to constantly be changing hardware (computer, phone, tablet or other) for online studies
	Hay frecuentes actualizaciones en las redes computacionales en mi universidad o instituto	There are frequent upgrades in the computer networks at my university or college
**Conflict study-** **home (work-home)**	Las obligaciones de mis estudios online interfieren con mi vida familiar y de hogar	The demands of my work interfere with my home and family life.
	La cantidad de tiempo que necesito para mis estudios online me dificulta cumplir con mis responsabilidades familiares	The amount of time my job takes up makes it difficult to fulfill family responsibilities
	Las cosas que quiero hacer en mi casa, no puedo hacerlas por las obligaciones que mis estudios online me imponen	Things I want to do at home do not get done because of the demands my job puts on me
	Mis estudios online generan una presión que me hace difícil cumplir con mis deberes familiares	My job produces strain that makes it difficult to fulfill my family or private duties.
	Debido a los deberes de mis estudios online, tengo que hacer cambios en mis actividades familiares planificadas	Due to work-related duties, I have to make changes to my plans for family activities.
**Conflict home-** **Study (home-work)**	Las demandas de mi familia interfieren con las actividades relacionadas a mis estudios online	The demands of my family or spouse/partner interfere with work-related activities
	He tenido que posponer actividades relacionadas a mis estudios online debido a las demandas de tiempo de mi familia	I have to put off doing things at work because of demands on my time at home
	No puedo hacer todas las cosas de mis estudios online que me gustaría, debido a las demandas de mi familia	Things I want to do at work don’t get done because of the demands of my family or spouse/partner
	Mi vida familiar interfiere con las responsabilidades que tengo en mis estudios online, como entrar a clases a tiempo o cumplir con mis tareas y trabajos.	My home life interferes with my responsibilities at work such as getting to work on time, accomplishing daily tasks, and working overtime
	Las presiones familiares interfieren con mi capacidad para rendir en mis estudios online.	Family-related strain interferes with my ability to perform job- related duties.
**Satisfaction with university life**	En la mayoría de los aspectos, mi actual vida en la Universidad/Instituto se acerca a mi ideal	In most ways my life at ** University is close to my ideal
	Las condiciones actuales de mi vida en la Universidad/Instituto son excelentes	The conditions of my life at ** University are excellent
	Actualmente, he obtenido las cosas importantes que buscaba en la Universidad/Instituto	So far I have gotten the important things I want at ** University
	Actualmente, estoy satisfecho con mi vida en la Universidad/Instituto	I am satisfied with my life at ** University.

**Table A2:** Descriptive results for the main indicators, averaged over the sample.

	Gen (M = 1)	N	Mean	Median	SD	Minimum	Maximum
**Age**	1	102	22.843	23.000	2.370	18	29
	2	98	22.694	22.000	3.533	18	51
**MP-Education**	1	102	0.232	0.333	0.247	0.00	1.000
	2	98	0.272	0.333	0.289	0.00	1.000
**MP-Health**	1	102	0.209	0.333	0.234	0.00	1.000
	2	98	0.276	0.333	0.253	0.00	1.000
**MP-Working**	1	102	0.248	0.333	0.272	0.00	1.000
	2	98	0.204	0.333	0.223	0.00	0.667
**MP-Networking**	1	102	0.154	0.000	0.218	0.00	1.000
	2	98	0.122	0.000	0.194	0.00	0.667
**MP-Housing**	1	102	0.337	0.333	0.246	0.00	0.667
	2	98	0.401	0.333	0.262	0.00	1.000
**TD-Overload**	1	102	3.422	3.500	0.771	1.40	5.000
	2	98	3.729	3.800	0.801	1.00	5.000
**TD-Invasion**	1	102	3.855	4.000	0.883	1.00	5.000
	2	98	4.125	4.250	0.879	1.00	5.000
**TD-Complexity**	1	102	2.277	2.500	0.898	1.00	4.250
	2	98	2.594	2.500	1.013	1.00	5.000
**TD-Uncertainty**	1	102	2.797	2.750	0.827	1.00	4.500
	2	98	2.990	3.000	0.816	1.25	5.000
**Satisfaction**	1	102	3.221	3.125	1.574	1.00	7.000
	2	98	2.990	2.625	1.580	1.00	7.000
**Study-Home C**	1	102	3.476	3.600	0.915	1.40	5.000
	2	98	3.608	3.800	1.108	1.00	5.000
**Home-study C**	1	102	2.739	2.600	1.007	1.00	5.000
	2	98	2.943	3.000	1.062	1.00	5.000

**Table A3:** Assessment of the measurement model for the first-order constructs.

Construct	Weights	Loads	VIF	CA	CR	AVE
**Home-study conflict (HSC)**				**0.901**	**0.927**	**0.717**
HSC_1 ← HSC	0.242 ***	0.844 ***	2.279			
HSC_2 ← HSC	0.217 ***	0.829 ***	2.323			
HSC_3 ← HSC	0.242 ***	0.875 ***	2.812			
HSC_4 ← HSC	0.225 ***	0.847 ***	2.386			
HSC_5 ← HSC	0.255 ***	0.838 ***	2.196			
**Satisfaction with life university (SA)**				**0.913**	**0.939**	**0.793**
SA-1 ← SA	0.270 ***	0.878 ***	2.748			
SA-2 ← SA	0.308 ***	0.887 ***	2.603			
SA-3 ← SA	0.251 ***	0.864 ***	2.634			
SA-4 ← SA	0.293 ***	0.932 ***	4.173			
**Study-home conflict (SHC)**				**0.900**	**0.926**	**0.714**
SHC_1 ← SHC	0.194 ***	0.796 ***	2.014			
SHC_2 ← SHC	0.244 ***	0.897 ***	3.259			
SHC_3 ← SHC	0.280 ***	0.866 ***	2.538			
SHC_4 ← SHC	0.219 ***	0.850 ***	2.471			
SHC_5 ← SHC	0.244 ***	0.813 ***	2.004			
**Techno-distress (TD)**						
TD-CO-1 ← CO	0.232 ***	0.865 ***	2.937			
TD-CO-1 ← TD	0.089 ***	0.645 ***	3.117			
TD-CO-2 ← CO	0.260 ***	0.890 ***	3.443			
TD-CO-2 ← TD	0.102 ***	0.723 ***	3.663			
TD-CO-3 ← CO	0.254 ***	0.848 ***	2.584			
TD-CO-3 ← TD	0.100 ***	0.705 ***	2.803			
TD-CO-4 ← CO	0.209 ***	0.737 ***	1.718			
TD-CO-4 ← TD	0.079 ***	0.580 ***	1.641			
TD-CO-5 ← CO	0.237 ***	0.837 ***	2.376			
TD-CO-5 ← TD	0.091 ***	0.659 ***	2.627			
TD-IN-1 ← IN	0.303 ***	0.771 ***	1.604			
TD-IN-1 ← TD	0.104 ***	0.632 ***	1.970			
TD-IN-2 ← IN	0.263 ***	0.745 ***	1.849			
TD-IN-2 ← TD	0.083 ***	0.549 ***	1.618			
TD-IN-3 ← IN	0.308 ***	0.883 ***	2.764			
TD-IN-3 ← TD	0.101 ***	0.642 ***	2.989			
TD-IN-4 ← IN	0.336 ***	0.888 ***	2.697			
TD-IN-4 ← TD	0.113 ***	0.702 ***	2.978			
TD-OL-1 ← OL	0.231 ***	0.666 ***	1.679			
TD-OL-1 ← TD	0.084 ***	0.551 ***	1.447			
TD-OL-2 ← OL	0.263 ***	0.766 ***	1.697			
TD-OL-2 ← TD	0.098 ***	0.627 ***	1.794			
TD-OL-3 ← OL	0.282 ***	0.777 ***	1.693			
TD-OL-3 ← TD	0.104 ***	0.672 ***	2.035			
TD-OL-4 ← OL	0.277 ***	0.735 ***	1.565			
TD-OL-4 ← TD	0.101 ***	0.660 ***	1.845			
TD-OL-5 ← OL	0.297 ***	0.744 ***	1.604			
TD-OL-5 ← TD	0.107 ***	0.708 ***	2.036			
TD-UN-1 ← TD	0.060 ***	0.415 ***	1.399			
TD-UN-1 ← UN	0.332 ***	0.679 ***	1.239			
TD-UN-2 ← TD	0.060 ***	0.402 ***	1.847			
TD-UN-2 ← UN	0.322 ***	0.735 ***	1.629			
TD-UN-3 ← TD	0.089 ***	0.558 ***	1.482			
TD-UN-3 ← UN	0.447 ***	0.733 ***	1.198			
TD-UN-4 ← TD	0.051 ***	0.360 ***	1.617			
TD-UN-4 ← UN	0.288 ***	0.732 ***	1.697			
**Multidimensional poverty (MP)**						
MP-EDb-1 → ED	0.672 **	0.737 ***	1.009			
MP-EDb-1 → MP	0.187 **	0.386**	1.077			
MP-EDb-2 → ED	0.679 **	0.744 ***	1.009			
MP-EDb-2 → MP	0.191 **	0.390 **	1.122			
MP-HEb-2 → HE	0.360 *	0.461 **	1.013			
MP-HEb-2 → MP	0.147 *	0.345 **	1.117			
MP-HEb-3 → HE	0.893 ***	0.934 ***	1.013			
MP-HEb-3 → MP	0.354 ***	0.699 ***	1.364			
MP-HOb-1 → HO	0.356 ns	0.452 *	1.011			
MP-HOb-1 → MP	0.120 ns	0.293 *	1.137			
MP-HOb-3 → HO	0.897 ***	0.935 ***	1.011			
MP-HOb-3 → MP	0.301 ***	0.607 ***	1.178			
MP-NTb-2 → NT	0.669**	0.778 ***	1.030			
MP-NTb-2 → MP	0.161 *	0.365**	1.133			
MP-NTb-3 → NT	0.638 *	0.752**	1.030			
MP-NTb-3 → MP	0.165 *	0.353**	1.177			
MP-WOb-1 → WO	0.856 ***	0.930 ***	1.040			
MP-WOb-1 → MP	0.268 ***	0.630 ***	1.279			
MP-WOb-2 → WO	0.376 *	0.543 **	1.040			
MP-WOb-2 → MP	0.138 *	0.368 **	1.193			

*: *p* < 0.05; **: *p* < 0.01; ***: *p* < 0.001; ns: non-significant. VIF: Variance inflation factor. CA: Cronbach’s alpha. CR: Composite reliability. AVE: Average variance extracted.

**Table A4:** Assessment of the measurement model for the second-order constructs.

Technostress (TD)	Loads	CA	CR	AVE
TD → CO	0.794 ***	0.772	0.855	0.598
TD → IN	0.770 ***			
TD → OL	0.876 ***			
TD → UN	0.634 ***			
**Multidimensional poverty (MP)**	**Weights**			
ED → MP	0.278 ***			
HE → MP	0.405 ***			
HO → MP	0.332 ***			
NT → MP	0.245 ***			
WO → MP	0.324 ***			

*: *p* < 0.05; **: *p* < 0.01; ***: *p* < 0.001; ns: non-significant. CA: Cronbach’s alpha. CR: Composite reliability. AVE: Average variance extracted.

**Table A5:** Heterotrait-Monotrait Ratio for assessment of the divergent validity.

	CO	HSC	IN	OL	SA	SHC
HSC	0.274					
IN	0.402	0.320				
OL	0.628	0.363	0.832			
SA	0.259	0.202	0.486	0.488		
SHC	0.257	0.449	0.641	0.617	0.327	
UN	0.628	0.361	0.329	0.512	0.141	0.190

HSC: Home-study conflict; IN: Techno-invasion; OL: Techno-overload; SA: Satisfaction with university life; SHC: Study-home conflict; UN: Techno-uncertainty.

**Table A6:** Validity, and reliability estimates for the second-order model.

Construct	CA	CR	AVE	HTMT		
				HSC	SA	SHC
HSC	0.901	0.927	0.717			
SA	0.913	0.939	0.793	0.202		
SHC	0.900	0.926	0.714	0.449	0.327	
TS	0.772	0.846	0.585	0.449	0.452	0.577

CA: Cronbach’s alpha; CR: Composite reliability; AVE: Average variance extracted; HTMT: Heterotrait-Monotrait Ratio. HSC: Home-study conflict; SA: Satisfaction with university life; SHC: Study-home conflict; TS: Technostress.
